# The relationship between symptoms and blood pressure during maintenance hemodialysis

**DOI:** 10.1111/hdi.12306

**Published:** 2015-05-07

**Authors:** David J Meredith, Christopher W Pugh, Sheera Sutherland, Lionel Tarassenko, Jacqueline Birks

**Affiliations:** 1 The Nuffield Department of Clinical Medicine, University of OxfordOxford, UK; 2 Oxford Kidney Unit, Churchill HospitalOxford, UK; 3 Institute of Biomedical Engineering, University of OxfordOxford, UK; 4 Medical Statistics, Centre for Statistics in MedicineOxford, UK

**Keywords:** Blood pressure, hemodialysis, intradialytic hypotension, symptoms

## Abstract

Intradialytic hypotension (IDH) is a detrimental complication of maintenance hemodialysis, but how it is defined and reported varies widely in the literature. European Best Practice Guideline and Kidney Disease Outcomes Quality Initiative guidelines require symptoms and a mitigating intervention to fulfill the diagnosis, but morbidity and mortality outcomes are largely based on blood pressure alone. Furthermore, little is known about the incidence of asymptomatic hypotension, which may be an important cause of hypoperfusion injury and impaired outcome. Seventy-seven patients were studied over 456 dialysis sessions. Blood pressure was measured at 15-minute intervals throughout the session and compared with post-dialysis symptom questionnaire results using mixed modeling to adjust for repeated measures in the same patient. The frequency of asymptomatic hypotension was estimated by logistic regression using a variety of commonly cited blood pressure metrics that describe IDH. In 113 sessions (25%) where symptoms were recorded on the questionnaire, these appear not to have been reported to dialysis staff. When symptoms were reported (293 sessions [64%]), an intervention invariably followed. Dizziness and cramp were strongly associated with changes in systolic blood pressure (SBP), but not diastolic blood pressure. Nausea occurred more frequently in younger patients but was not associated with falls in blood pressure. Thresholds that maximized the probability of an intervention rather than a session remaining asymptomatic were SBP <100 mmHg or a 20% reduction in SBP from baseline. The probability of SBP falling to <100 mmHg in an asymptomatic session was 0.23. Symptoms are frequently not reported by patients who are hypotensive during hemodialysis, which leads to an underestimation of IDH if symptom-based definitions are used. A revised definition of IDH excluding patient-reported symptoms would be in line with literature reporting morbidity and mortality outcomes and include sessions in which potentially detrimental asymptomatic hypotension occurs.

## Introduction

Intradialytic hypotension (IDH) is a recognized complication of hemodialysis, but how it is defined and reported varies widely in the literature. Variation results from different blood pressure (BP) thresholds at which hypotension is deemed to occur and inconsistency whether associated symptoms such as nausea, vomiting, dizziness, and cramp are a prerequisite for a diagnosis of IDH.^1^ Some definitions go further and require the occurrence of both patient-reported symptoms and an intervention (the reduction of ultrafiltration [UF] rate or blood pump speed, tilting of the bed, or the administration of a saline bolus) to fulfill the diagnosis—European Best Practice Guidelines and Kidney Disease Outcomes Quality Initiative guidelines are explicit in this regard.^2^,^3^ Hypotension during dialysis results in myocardial regional wall motion abnormalities, myocardial stunning, progressive heart failure, and is an independent risk factor for mortality. However, the studies demonstrating morbidity and/or mortality associated with dialysis often do not specify the presence of symptoms or an intervention and define IDH based solely on a critical reduction in BP.^4^–^6^

Usual practice is only to measure BP before and after dialysis, unless the patient appears or feels unwell during the treatment. As such, the measurement of intradialytic BP, and any subsequent intervention, is initiated by patient-reported symptoms or overt signs of hypoperfusion. Although this provides a rationale for the triangulation between symptoms and hypotension used by the guidelines, it results in a disconnection between the standard definition of IDH and that used in published outcome studies. The sequence of events leading to intradialytic measurement of BP also explains why little is known about the incidence of asymptomatic hypotension in the maintenance dialysis population. Establishing the frequency of asymptomatic hypotension is of importance because otherwise patients with recurrent asymptomatic hypotensive events and potentially detrimental cumulative subclinical hypoperfusion injury go undetected.

This study addresses these issues by (i) exploring the propensity for a patient to report symptoms during dialysis; (ii) outlining the association between nausea, dizziness, cramp, and intradialytic BP; and (iii) establishing the frequency of asymptomatic hypotension (defined using commonly cited BP thresholds) in our routine outpatient, largely Caucasian hemodialysis population, taking their normal prescription antihypertensives. Our data lead us to suggest an evidence-based guideline for the diagnosis of IDH.

## Materials and Methods

### Subjects

Following ethics approval, all adult hemodialysis patients within one outpatient dialysis unit of a tertiary referral center in the United Kingdom were invited to take part in this prospective observational study. In total, 77 of 94 eligible patients agreed to participate.

### Prestudy health survey

The study period coincided with an annual unit-wide quality-of-life assessment as it was recognized that a variety of physical, emotional, and health perception factors may impact symptom reporting. The Short Form “SF-36” health survey is a multipurpose questionnaire and outcomes are widely cited and validated for patients with end-stage renal disease.^7^–^9^

Of 77 study participants, 50 respondents completed SF-36 questionnaires and their scores were compared with 73 patients across the unit’s entire dialysis population external to the study to establish whether or not our results were likely to be representative of a wider dialysis population, including those in regional satellite units.

### Study protocol

The dialysis environment, the dialysis prescription, and the patients’ medication were not changed for the purposes of this study. Hemodialysis was conducted using Dialog+ Machines (B. Braun, Melsungen, Germany), incorporating Critline (Hema Metrics, Boston, MA, USA) relative blood volume monitors, and high-flux dialyzers. Room temperature was 22°C, and dialysate temperature ranged between 36.5°C and 37°C. Dialysate solutions were bicarbonate based and conductivity ranged between 13.8 and 14.3 mS/cm.

Four patients were studied simultaneously in line with the 4:1 patient-to-nurse ratio common to most UK outpatient hemodialysis units. BP was measured every 15 minutes for all study participants during the observational period, unless clinical judgment mandated extra-assessments. Systolic blood pressure (SBP), diastolic blood pressure (DBP), and mean arterial pressure (MAP) measurements were taken on the nonfistula arm using a cuff-oscillometric device. Both patient and dialysis nurse were blinded to the BP and Critline interface display on the dialysis machine monitor unless clinical concerns warranted closer hemodynamic monitoring.

The noninvasive BP (NIBP) recordings and session-specific data (ultrafiltration volume and blood pump speed) were stored in B. Braun’s Nexadia software (B. Braun, Melsungen, Germany) and downloaded after each session for subsequent analysis. Time-synchronized Critline data were stored in the device’s internal memory, and then also downloaded after dialysis. Data from the Nexadia and Critline databases were assimilated into session-specific files within a unified database using MatLab R2012a (MathWorks, Inc., Natick, MA, USA) for further analysis. Total ultrafiltration volume for each treatment was assessed and also expressed as a percentage of the patient’s dry weight. Using Critline data, the largest percentage increase in hematocrit concentration was used as a surrogate for the lowest relative blood volume achieved for each session (expressed as a percentage reduction from the start of dialysis).

After assigning the BP reading immediately following bleed-out into the extracorporeal circuit as “baseline,” the following commonly cited descriptors of IDH were defined for SBP, DBP, and MAP for each session: (i) the lowest recorded value (“minimum”) for the session (mmHg); (ii) the difference (“delta”) between the baseline BP and minimum BP, i.e., Baseline − Minimum (mmHg); (iii) delta as a percentage of baseline, i.e., (Baseline − Minimum)/Baseline*100 (%); (iv) the difference between two consecutive 15-minute measurements (“Delta BP over 15 minutes”), i.e., BP_T-15_ − BP_T_ (mmHg), where T = time of current BP measurement in minutes; (v) delta BP over 15 minutes as a percentage of the baseline value, i.e., (BP_T-15_ − BP_T_)/Baseline*100 (%).

### Post-dialysis symptom questionnaire

To minimize the impact of the study on a patient’s propensity to report symptoms, participants were asked to complete a questionnaire immediately following dialysis to describe the nature and severity of symptoms experienced during the preceding treatment. The questionnaire included symptoms with which the patients were familiar and are considered synonymous with a reduction in blood volume or BP, such as nausea, dizziness,^10^ and cramps.^11^ Each of the specified symptoms was stratified by severity: none, trivial, mild, moderate, and severe and scored 0, 1, 2, 3, or 4, respectively. Nursing documentation was collated and transcribed for each study session so that it could be compared with patients’ post-dialysis questionnaire responses.

### Categorization of the study sessions and assignment of groups for analysis

The study sessions were divided into three categories according to post-dialysis symptom questionnaire responses and/or any intervention received by the patient during dialysis: First, sessions scoring zero for all symptoms (nausea, dizziness, and cramp) were assigned to the “asymptomatic” group. Second, sessions scoring one or more for any of these symptoms but not receiving an intervention were assigned to the “symptomatic” group. Finally, an “intervention” group was created for sessions in which symptoms or signs of hypoperfusion (e.g., excessive sweating, sudden change in skin tone/cyanosis, or cognitive impairment/reduced consciousness) prompted an immediate intervention such as the reduction of ultrafiltration rate or blood pump speed, tilting of the bed, or the administration of a saline bolus. Interventions due to vascular access problems or clotting of the extracorporeal circuit were excluded from the analysis.

### Statistical analysis

Mean (standard deviation) or median (interquartile range) were used as descriptive statistics in the analysis of the study subjects’ demographic data. Analyses of SF-36 scores for the study patients and nonstudy patients were conducted using nonparametric Mann-Whitney U tests as the data for the eight SF-36 outcome measures were not normally distributed.

Repeated measurements during a dialysis session over repeated sessions, for more than one patient, represent a multilevel data structure. The hierarchical nature of our three-level data was taken into account using mixed (fixed and random errors) modeling and standard errors/confidence intervals were adjusted accordingly. The mixed-model analyses were carried out independently by a medical statistician. Two-level analyses of the functions of SBP, DBP, and MAP incorporated covariates at the session level (type of session, total UF, UF adjusted for weight, and maximum decrease in relative blood volume for the session) while adjusting for repeat sessions or the same patient. Symptom scores were dichotomized, i.e., symptom score of 0 (asymptomatic) compared with symptoms of any severity (1–4). Covariates at the patient level included age, gender, months on dialysis, whether a patient had type 1 or type 2 diabetes, and the number of antihypertensives prescribed.

In order to obtain a probability of a patient from a particular group reaching a predetermined BP threshold, each BP value for every session was coded as 0 if it was below the threshold or 1 if it was above the threshold. This binary variable at the session level was also analyzed using logistic regression in a generalized mixed model. Findings were reported as significant at the 5% level (P < 0.05).

## Results

Seventy-seven patients were studied over a total of 456 dialysis sessions. Patient characteristics in Table 1 reflect our dialysis population, which is predominately Caucasian and male with a median age of 65 years. Of the 77 participants, 31 (40.3%) were diabetic. Table 2 shows nonsignificant differences in both the demographics and responses for each of eight categories of the SF-36 questionnaire when the 50 study respondents were compared with 73 patients dialyzing in three of our regional satellite units. Each patient was studied for a mean of 5.9 sessions (range 1–19) over 12 months.

**Table 1:** Patient characteristics

	All patients (n = 77)	%
Male : Female	55:22	
Age, years median (IQR)	65 (51–75)	
RRT vintage, months median (IQR)	30 (14–66)	
HD vintage, months median (IQR)	26 (11–49)	
Ethnicity		
White	68	88.3
Black	3	3.9
South Asian	3	3.9
Other	3	3.9
Primary diagnosis		
Glomerulonephritis	24	31.2
Diabetic nephropathy	16	20.8
Hypertensive/renovascular	9	11.7
Pyelonephritis/reflux	5	6.5
Polycystic disease	4	5.2
Renal dysplasia	3	3.9
Other or unknown	16	20.8
Smoker	15	19.5
Diabetes		
Type 1	6	7.8
Type 2	25	32.5
Ischemic heart disease	30	39.0
Left ventricular hypertrophy	16	20.8
Listed for renal transplant	19	24.7
Antihypertensive medication		
Angiotensin converting enzyme inhibitors (ACEi)	16	20.8
Aldosterone receptor blockers (A2RB)	16	20.8
Beta-blocker	29	37.7
Alpha-blocker	12	15.6
Calcium channel blocker	26	33.8
Other	8	10.4
Antihypertensives per patient		
0	23	29.9
1	14	18.2
2	31	40.3
3	5	6.5
4	4	5.2

HD = hemodialysis; IQR = interquartile range; RRT = Renal Replacement Therapy.

**Table 2:** Results from a unit-wide SF-36 health survey

	SF-36 respondents in study	Nonstudy SF-36 respondents
Number of responses	50 of 77	73 of 129
Age (years)	67 (51–77)	71 (52–77)
Months on HD	24 (12–46)	26 (12–49)
Male: Female	34:16	46:27
	SF-36 health survey score	
Respondents in study	Nonstudy respondents	
Physical functioning	30 (6–50)	25 (5–45)
Role limitations due to physical health	0 (0–46)	0 (0–25)
Role limitations due to emotional problems	33 (0–100)	33 (0–100)
Energy/fatigue	33 (16–45)	35 (15–55)
Emotional well-being	68 (56–80)	72 (52–88)
Social functioning	69 (50–84)	63 (50–88)
Pain	55 (43–78)	55 (23–90)
General health	40 (33–56)	38 (25–34)

Results are expressed as median (interquartile range). Comparisons were nonsignificant using a Mann-Whitney test at the 5% level. (i) Physical functioning (0 = very poor physical functioning to 100 = excellent physical functioning); (ii) role limitations due to physical function (0 = no limitation to 100 = extremely limited); (iii) role limitation due to personal or emotional problems (0 = no limitation to 100 = extremely limited); (iv) energy/fatigue (0 = low energy/high fatigue to 100 = high energy/low fatigue); (v) emotional well-being (0 = very poor emotional well-being to 100 = excellent emotional well-being); (vi) social functioning (0 = very poor social functioning to 100 = excellent social functioning); (vii) bodily pain (0 = no pain to 100 = continuous pain); and (viii) general health perceptions (0 = general health perceived as very poor to 100 = general health perceived as excellent).

HD = hemodialysis.

### Symptom reporting

For the 298 sessions assigned to the asymptomatic group on the basis of the post-dialysis questionnaire, there was the expected absence of patient-reported symptoms in the nurses’ notes. However, all 113 sessions assigned to the symptomatic group on the basis of the post-dialysis symptom questionnaire were also without corresponding documentation of the patient’s symptoms in the nurses’ notes so would not have triggered extra BP measurements during routine care. Conversely, 38 of the 45 intervention sessions accurately documented patient-reported symptoms, and the remaining seven sessions documented that an intervention had occurred due to signs of hypoperfusion in an otherwise asymptomatic patient.

### Relationship of symptoms to patient-specific and session-specific covariates

Table 3 shows that the percentage of sessions in which the patient experienced nausea, dizziness, or cramp was 22.1%, 12.3%, and 7.5%, respectively. For each symptom, the frequency of occurrence was inversely related to the graded severity. The mixed-model logistic regression analysis showed that nausea was not related to any function of BP, but was significantly associated with a lower age (odds ratio for 10 years increment in age of 0.52 [0.30–0.91], P = 0.023). Dizziness was significantly associated with session minimum BP (odds ratio for 10 mmHg increase in minimum BP was 0.75 [0.62–0.91], P = 0.004 for SBP and 0.68 [0.52–0.89], P = 0.005 for MAP), but not patient-level covariates. Cramps were significantly associated with baseline SBP − minimum SBP difference (odds ratio for 10 mmHg delta SBP was 1.16 [1.00–1.36], P = 0.05) but not associated with changes in MAP or patient-level covariates.

**Table 3:** Number of sessions reporting symptoms categorized by symptom severity

Group	Severity score	Nausea	Dizziness	Cramp
Asymptomatic (n = 298)	0 (asymptomatic)	298	298	298
Symptomatic (n = 113)	0 (for specified symptom only)	84	67	39
	1 (trivial)	14	22	38
	2 (mild)	9	20	18
	3 (moderate)	3	2	16
	4 (severe)	2	1	2
	Not recorded	1	1	0
Intervention (n = 45)	0 (asymptomatic)	38	34	18
	1 (trivial)	3	4	7
	2 (mild)	1	3	9
	3 (moderate)	1	3	5
	4 (severe)	1	1	6
	Not recorded	1	0	0
	Total	456	456	456
	% Sessions reporting symptom	7.5	12.3	22.1

Table 4 shows a nonsignificant difference between the total UF volume of the asymptomatic and intervention groups (reflecting the reduction in UF rate and/or provision of a saline bolus) but a significantly increased UF volume in the symptomatic group compared with the asymptomatic group. However, the maximum decrease in relative blood volume achieved during dialysis was not significantly different between the three groups. Baseline BP was equivalent among the asymptomatic, symptomatic, and intervention groups, but symptomatic and intervention sessions experienced significantly lower minimum SBPs than the asymptomatic group. These findings remain highly significant when the delta (baseline − minimum) BP is adjusted for baseline. Moreover, logistic regression analysis showed the odds of an intervention increased significantly for every 10 mmHg increment in delta SBP (1.41 [1.17–1.70], P < 0.0001). These findings were generally not true of DBPs apart from significant difference between asymptomatic and intervention DBP (23% vs. 29%, P < 0.01). Maximum fall in SBP between consecutive measurements (i.e., delta over 15 minutes) was significantly lower in both symptomatic and intervention group; however, delta SBP over 15 minutes, delta DBP over 15 minutes, and delta MAP over 15 minutes were all nonsignificant when adjusted for baseline values. The rate of change of SBP and DBP over the first hour of dialysis in the three groups was not significantly different. There were no significant changes in any reported outcome in post-hoc analyses when the intervention session data were censored at the time of the (first) intervention.

**Table 4:** Mean value of variables for each group adjusted for repeat measurements on the same patient

	Asymptomatic (n = 298)	Symptomatic (n = 113)	Intervention (n = 45)
UF volume (L)	2.23 (0.11)	2.43 (0.13), P = 0.02	2.24 (0.14), P = 0.91
Adjusted UF volume (% of dry weight)	3.00 (0.15)	3.24 (0.17), P = 0.03	2.99 (0.19), P = 0.98
Lowest blood volume (% reduction)	−9.32 (0.45)	−9.69 (0.64), P = 0.57	−8.53 (0.85), P = 0.36
Baseline SBP (mmHg)	139.5 (2.6)	139.4 (3.1), P = 0.97	141.1 (3.7), P = 0.62
Minimum SBP (mmHg)	111.2 (2.3)	104.8 (2.8), P = 0.006	102.4 (3.4), P = 0.003
Delta SBP (% of baseline SBP)	0.20 (0.01)	0.24 (0.02), P = 0.007	0.27 (0.02), P < 0.001
Delta SBP over 15 min (mmHg)	26.1 (1.2)	27.5 (1.3), P < 0.52	29.4 (1.3), P < 0.63
Delta SBP over 15 min (% of baseline SBP)	0.19 (0.01)	0.2 (0.001), P = 0.70	0.2 (0.02), P = 0.8
Baseline DBP (mmHg)	78.2 (1.8)	78.4 (2.1), P = 0.90	82.0 (2.5), P = 0.08
Minimum DBP (mmHg)	59.4 (1.5)	59.7 (1.8), P = 0.29	57.5 (2.2), P = 0.29
Delta DBP (% of baseline DBP)	0.23 (0.01)	0.25 (0.02), P = 0.21	0.29 (0.02), P = 0.01
Delta DBP over 15 min (mmHg)	20.6 (1.3)	20.7 (1.8), P = 0.98	22.9 (2.3), P = 0.32
Delta DBP over 15 min (% of baseline DBP)	0.28 (0.02)	0.27 (0.03), P = 0.73	0.27 (0.04), P = 0.81
Baseline MAP (mmHg)	98.6 (1.8)	98.7 (2.2), P = 0.94	101.7 (2.6), P = 0.17
Minimum MAP (mmHg)	79.1 (1.9)	75.4 (2.0), P = 0.023	74.3 (2.8), P = 0.021
Delta MAP (% of baseline MAP)	0.19 (0.01)	0.23 (0.02), P = 0.021	0.27 (0.02), P < 0.001
Delta MAP over 15 min (mmHg)	18.8 (1.1)	20.4 (1.5), P = 0.29	22.9 (2.3), P = 0.32
Delta MAP over 15 min (% of baseline MAP)	0.19 (0.01)	0.21 (0.02), P = 0.32	0.2 (0.02), P = 0.90
Rate of SBP change in first hour (mmHg/h)	−7.1 (0.5)	−7.1 (0.5), P = 0.76	−7.2 (0.5), P = 0.08
Rate of DBP change in first hour (mmHg/h)	−3.9 (0.2)	−3.8 (0.2), P = 0.76	−4.0 (0.2), P = 0.16

Results are expressed as mean (standard error) and P value for comparison with the asymptomatic group. Symptomatic vs. intervention comparisons were all nonsignificant at the 5% level.

DBP = diastolic blood pressure; MAP = mean arterial blood pressure; SBP = systolic blood pressure; UF = ultrafiltration.

**Figure 1:**
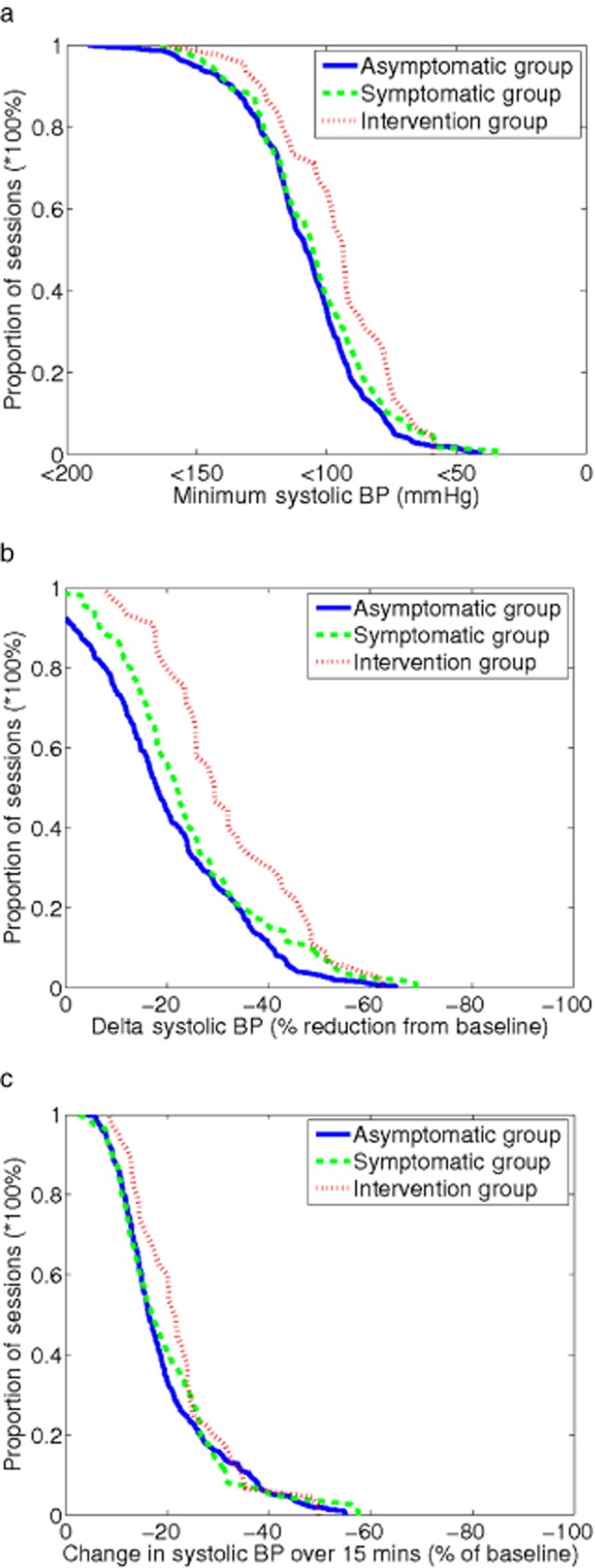
Cumulative frequency profiles for various systolic blood pressure (SBP) metrics used to describe intradialytic hypotension in asymptomatic, symptomatic, and intervention groups. Panel (a) shows lowest (nadir) SBP during dialysis. Panel (b) shows delta SBP (Baseline SBP − Nadir SBP) as a percentage of baseline SBP. Panel (c) shows fall in SBP between two consecutive measurements as a percentage of baseline SBP.

**Figure 2:**
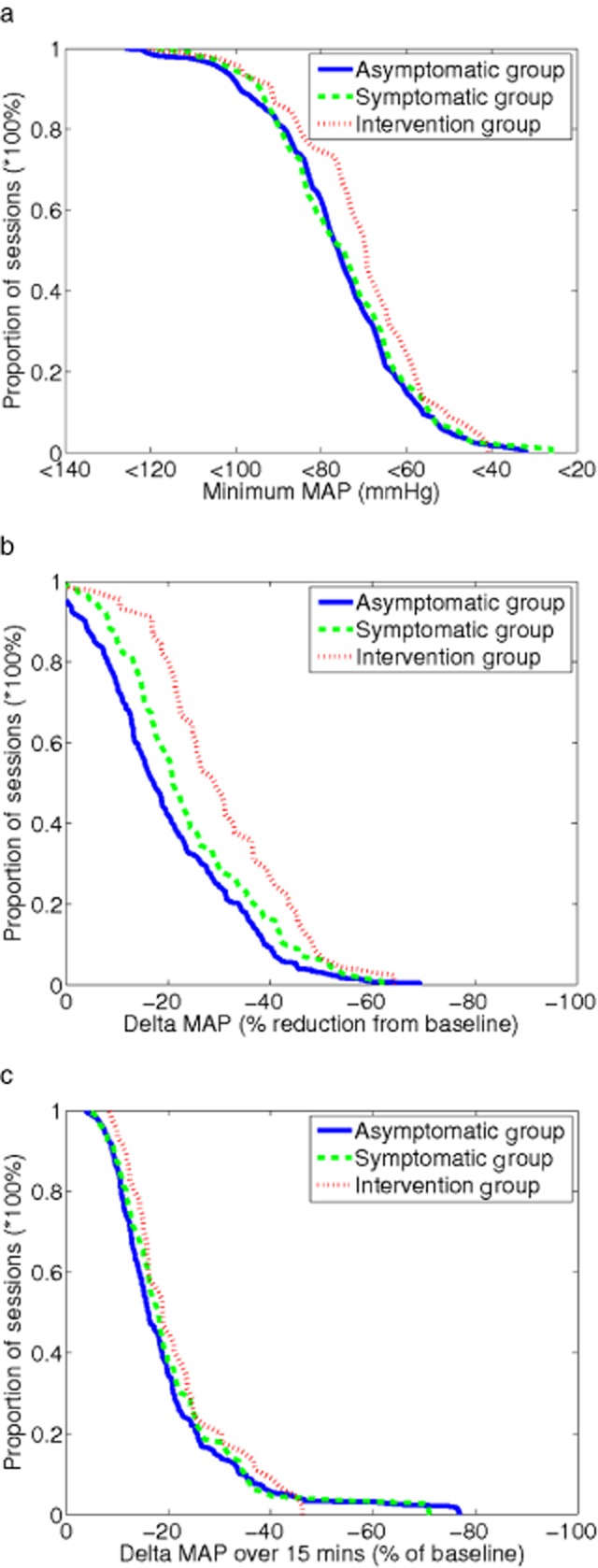
Cumulative frequency profiles for various mean arterial blood pressure (MAP) metrics used to describe intradialytic hypotension in asymptomatic, symptomatic, and intervention groups. Panel (a) shows lowest (nadir) MAP during dialysis. Panel (b) shows delta MAP (Baseline MAP − Nadir MAP) as a percentage of baseline MAP. Panel (c) shows fall in MAP between two consecutive measurements as a percentage of baseline MAP.

Figure 1a shows that a minimum SBP <100 mmHg is the threshold with greatest discrimination between asymptomatic and intervention sessions (35% vs. 64% respectively, or probability of SBP <100 mmHg = 0.23 vs. 0.55, respectively [P = 0.003], when analyzed using logistic regression in a generalized mixed model). Likewise, Figure 1b shows maximal separation between the proportion of asymptomatic and intervention groups occurred when delta SBP was 25% of baseline (maximum discrimination was estimated at 20% using mixed-model logistic regression when repeated measurements from the same patient were taken into account). Figure 1c shows the delta SBP over 15 minutes as a percentage of baseline SBP; differences between groups were not statistically significant. Figure 2 illustrates comparable trends for MAP, but here the ability to discriminate between groups is less than that shown in Figure 1 for SBP.

## Discussion

It is clear that for purposes of clinical audit or research, a standardized yet evidence-based approach to the definition of IDH is required if intradialytic adverse events are to be compared within a particular dialysis unit over time, or between units or patient groups inter-nationally. In this study, we present results that may help to refine the current definitions.

First, we consider symptom reporting. When nausea, dizziness, or cramp were reported to the nurses and documented during dialysis (38 of 151, 25.2% sessions with symptoms), an appropriately documented intervention inevitably followed. However, in all 113 of the sessions labeled symptomatic purely on the basis of the post-dialysis questionnaire, an intervention did not occur (113 of 151, 74.8%). Possible reasons are (i) symptoms were not reported to the dialysis staff by the patient during the session. This could represent a research effect, with patients over-reporting symptom severity on the post-dialysis questionnaire that would have otherwise been dismissed as (less than) trivial during dialysis. Alternatively, patients may have experienced nontrivial symptoms but then either opted not to report these because symptoms were considered inevitable or unlikely to be improved with nursing intervention, or the patient had not been given the opportunity to report symptoms to a member of staff at the time of occurrence. (ii) Symptoms were reported by the patient but these were deemed not to warrant an intervention by nursing staff, and this decision was not documented. This may be the case for symptoms graded as trivial or mild by the patient, but symptoms reported as moderate or severe (there are 25 such reports in the symptomatic group in Table 3) would have been expected to result in an intervention to reduce severity, regardless of the BP. (iii) The patient reported symptoms that warranted intervention during dialysis but these were not appropriately documented or acted upon. We consider this third scenario unlikely, but cannot exclude it.

The disconnection in this group of patients between the occurrence of symptoms and the reporting of these symptoms during dialysis is important when contemporary definitions of IDH are considered. The under-reporting of symptoms in association with hypotension, as suggested in our study by patients in the symptomatic group reaching SBP levels <100 mmHg, could lead to a false reduction in IDH rates according to current guidelines, especially when it appears that the additional diagnostic requirement—an intervention—usually follows symptom reporting. Another potential source of inconsistency is that there is no universal agreement as to what symptoms should be deemed indicative of IDH. Various symptoms are commonly reported during dialysis that may sometimes be associated with hypotension (e.g., abdominal pain). We limited the selection of symptoms to those which we anticipated would have the highest sensitivity and specificity for hypotension; however, we accept that this pragmatic approach is a potential limitation in this study. Caplin et al. highlight variations in dialysis-associated symptoms attributable to gender and ethnicity, as well as unexplained inter-regional differences.^12^ Because our patients were predominantly male Caucasians, our study was not powered to make similar comparisons.

Our results showed a strong relationship between the intradialytic reduction of BP, dizziness, and cramp. However, this does not necessarily imply causation as potential confounders such as ultrafiltration rate, dialysate tonicity, and patient vs. dialysate temperature were not taken into account in our analyses. Of interest was our finding that nausea during dialysis was more likely to be a function of age than of BP, with younger patients reporting nausea more frequently than older patients.

Current definitions of IDH are based on changes in BP compared with baseline, rather than an absolute level of BP. In our study, the metric most strongly associated with dizziness and cramp appears to be SBP. The association between DBP and symptoms is poor, and the inferiority of MAP to SBP in the association with symptoms seems likely to be due to the DBP component of the MAP equation. A SBP <100 mmHg and/or reduction in SBP of 20% of baseline were the thresholds that maximized the probability that a session would result in an intervention. We included both relative and absolute descriptors of hypotension to take account of patients with chronic-sustained hypotension whose usual baseline SBP is <100 mmHg.

Of particular importance is our finding that a significant proportion of asymptomatic patients, treated with their routine antihypertensive medications, reach BP thresholds that potentially render them at risk of hypoperfusion injury. If the threshold of SBP <100 mmHg is considered, the probability of an asymptomatic patient reaching this threshold is 0.23 using mixed-model logistic regression, alternatively interpreted as systolic hypotension occurring in a quarter of all asymptomatic sessions. Given that there was a 15-minute interval between BP measurements during this study, it is probable that our results represent an underestimate of the true incidence of asymptomatic hypotension—i.e., it is possible that some patients experienced a reduction in BP that crossed this threshold but was followed by recovery within a 15-minute period. It is possible that this high incidence of hypotension is exacerbated by patients taking their routine antihypertensive medications on days when they are receiving dialysis, but this was not controlled in our study as we planned to capture standard practice in our unit. This study was not designed to address the mechanisms underlying symptomatic or asymptomatic hypotension and in future work it will be interesting to assess whether these categories of hypotension result from similar or dissimilar hemodynamic perturbations and the role of different antihypertensives in these effects.

The stipulation that symptoms are required in the definition of IDH may be considered by the authors of current guidelines to improve diagnostic accuracy, but what might be the rationale behind this association? The coupling of symptoms to BP for the diagnosis of IDH serves one of two functions: (i) either symptoms identify the occurrence of hypoperfusion in a particular individual at a BP threshold that may vary over time^13^ (i.e., reflects individual [organ] variation in the BP threshold at which hypoperfusion occurs and is, therefore, of limited utility when extrapolated to the wider population); or (ii) symptoms act as a surrogate for hypotension and are used to identify individuals that may be crossing a predefined population-based threshold (i.e., function as a “red flag” to trigger BP assessment). If the former, then identification of risk of intradialytic hypoperfusion injury should perhaps be solely symptom based and decoupled from a predefined BP threshold, and if the latter, then the risk of hypoperfusion injury should perhaps be defined against a population-based BP threshold and not symptoms.

When considering which of these distinctions is more appropriate, it is worth recalling that current, although limited, evidence indicates that there is a stronger association between symptoms and blood volume than symptoms and BP,^10^,^14^ and it is the absolute BP, rather than blood volume reduction or intradialytic symptoms *per se*, that is associated with morbidity and mortality in hemodialysis patients.^4^–^6^ Clearly, this unmasks practical difficulties: How do we establish when the dynamic fluctuations of intradialytic BP cross a predefined threshold in outpatients? Frequent NIBPs are one approach, but how frequent? In order to establish the frequency of asymptomatic hypotension, any interval would be suboptimal compared with continuous BP monitoring.

Although evidence suggests that detrimental outcomes are associated with low intradialytic BP, we cannot assume that interventions to decrease the occurrence of hypotension would reduce morbidity and mortality in asymptomatic patients. Further, if asymptomatic hypotension was readily identified, would the propensity to intervene lead to detrimental increases in interdialytic weight gain (IDWG) because of interrupted ultrafiltration and/or the overzealous administration of saline or would a timely intervention in response to subcritical hypotension improve dialysis adequacy and reduce IDWGs by avoiding interrupted dialyses caused by an unforeseen decline to critical hypotension?

To address these questions, future research should be focused on ways of continuously tracking intradialytic BP, establishing the mechanisms underlying hypotensive episodes and then assessing the outcomes of interventions designed to mitigate these mechanisms and prevent asymptomatic as well as symptomatic hypotension. We consider existing mortality and morbidity outcomes with the evidence presented here to suggest that a reduction in SBP by >20% from baseline to <100 mmHg (or a reduction in SBP by >20% alone in those with a baseline SBP <100 mmHg) is used to define those at risk of intradialytic hypoperfusion injury, regardless of symptoms.
